# Analysis of the Expression and Function of Key Genes in Pepper Under Low-Temperature Stress

**DOI:** 10.3389/fpls.2022.852511

**Published:** 2022-05-04

**Authors:** Bingqian Tang, Lingling Xie, Huiping Yang, Xiumin Li, Ying Chen, Xuexiao Zou, Feng Liu, Xiongze Dai

**Affiliations:** ^1^College of Horticulture, Hunan Agricultural University, Changsha, China; ^2^Longping Branch, Graduate School of Hunan University, Changsha, China; ^3^ERC for Germplasm Innovation and New Variety, Breeding of Horticultural Crops, Changsha, China; ^4^Key Laboratory for Vegetable Biology of Hunan Province, Changsha, China

**Keywords:** cold stress, ICE-CBF-COR, pepper, gene regulatory network, GO analysis

## Abstract

The mechanism of resistance of plants to cold temperatures is very complicated, and the molecular mechanism and related gene network in pepper are largely unknown. Here, during cold treatment, we used cluster analysis (*k*-means) to classify all expressed genes into 15 clusters, 3,680 and 2,405 differentially expressed genes (DEGs) were observed in the leaf and root, respectively. The DEGs associated with certain important basic metabolic processes, oxidoreductase activity, and overall membrane compositions were most significantly enriched. In addition, based on the homologous sequence alignment of *Arabidopsis* genes, we identified 14 positive and negative regulators of the ICE-CBF-COR module in pepper, including *CBF* and *ICE*, and compared their levels in different data sets. The correlation matrix constructed based on the expression patterns of whole pepper genes in leaves and roots after exposure to cold stress showed the correlation between 14 ICE-CBF-COR signaling module genes, and provided insight into the relationship between these genes in pepper. These findings not only provide valuable resources for research on cold tolerance, but also lay the foundation for the genetic modification of cold stress regulators, which would help us achieve improved crop tolerance. To our knowledge, this is the first study to demonstrate the relationship between positive and negative regulators related to the ICE-CBF-COR module, which is of great significance to the study of low-temperature adaptive mechanisms in plants.

## Introduction

Cold stress (at non-freezing temperatures) is one of the main types of abiotic stresses that affects the growth and geographical distribution of plants and causes death in severe cases. Cold stress, including at low (<20°C) and freezing (<0°C) temperatures (Chinnusamy et al., 2010; Sanghera et al., 2011), causes significant adverse effects on the growth and development of plants and restricts their distribution and productive capability (Chinnusamy et al., 2007; Ding et al., 2019, 2020). A low-temperature environment could induce the expression of cold-responsive genes (CORs) in plants. The function of these genes is to stabilize the membrane, to prevent freezing-induced damage (Thomashow, 1999).

The CBF-dependent low-temperature signaling pathway in plants has been discussed relatively thoroughly. The three CBF genes arranged in tandem on chromosome 4 of the *Arabidopsis thaliana* genome have been successively identified as important transcription factors located upstream of the CORs (Stockinger et al., 1997; Gilmour et al., 1998; Liu et al., 1998). The overexpression of CBF1–3 significantly enhanced the resistance of *Arabidopsis* plants to freezing temperatures, and the RNA sequence (RNA-seq) analysis of the *cbf1*/*cbf2*/*cbf3* mutants of *Arabidopsis* showed that *CBF* mutations affected 10–20% of CORs after their expression (Jia et al., 2016; Zhao et al., 2016). In addition, CBF has been identified in many other plant species, all of which have characteristic cold-induced response generating genes. Therefore, the CBF function is highly conserved in the low-temperature signaling pathway in plants and the relationship between CBFs is complex and deserves further exploration (Shi et al., 2018). The plant ICE-CBF-COR-signaling module was established (Chinnusamy et al., 2003; Hwarari et al., 2022). CBFs play a central role in cold acclimation (Kim Y. S. et al., 2015), and their level of transcription is significantly up-regulated by the CBF expression protein inducer (ICE). They also activate the expression of downstream CORs by binding to cis-elements in their promoters (Lee et al., 2005; Tang et al., 2020).

*Capsicum annuum* L. is a typical thermophilic vegetable, whose optimal growth occurs at a temperature range of 21–28. It lacks a cold acclimation mechanism and is very sensitive to cold stress during periods of growth. Genes such as *CaDHN4* (Zhang H. F. et al., 2019), *CaMADS* (Chen et al., 2019), and *CaPIF8*, which could increase low-temperature stress, have also been found to occur in peppers (Yang et al., 2021). Low-temperature stress causes a series of abnormal development-related phenomena in pepper, such as metabolic imbalance, reduced nutritional status, hindered female organ development, reduced number of pollen grains, and obstacles in the fertilization process, causing flowers and fruits to stop growing, fruits to be deformed, and even a lack of seeds. These phenomena significantly affect the quality and yield of peppers (Guo et al., 2015; Zhang et al., 2020).

Currently, there is a lack of systematic studies on the identification of CBF genes and CBF-related positive and negative regulatory factors in peppers. This analysis mainly focuses on the roots and leaves of pepper plants at six time points within 0.5–24 h under low-temperature stress conditions at 10°C. The expression of the whole set of genes was compared with that of the control. We constructed a gene regulation network for cold stress in pepper, and based on the CBF gene sequence in *A. thaliana* and currently available research, we identified the important genes that had positive and negative regulatory effects on the CBF genes in the entire pepper genome. We also assessed the expression of CBF signaling pathway-related genes after exposure to cold stress and the relationship between them in the regulatory network. This is the first study to discuss the relationship between positive and negative regulators related to the ICE-CBF-COR module. It is of great significance for the study of the low-temperature stress adaptive mechanism in pepper and discovery of stress resistance genes, which would facilitate the generation of more stress resistant plants for pepper breeding.

## Materials and Methods

### Plant Materials and Data Sources

In the previous study, total RNA was extracted from cold-treated leaves and roots of pepper (Liu et al., 2017). The elite breeding pepper (*Capsicum annuum*) line 6421 was selected from a long red pepper landrace grown widely in the West of the Xiangjiang River, in Hunan Province, China. It is resistant to anthracnose, bacterial spot, and bacterial wilt. Control plants were mock-treated with nutrient solution alone. Leaf and root tissues were collected from both treated and control plants at 0.5, 1, 3, 6, 12, and 24 h post-treatment (HPT).

### Differential Gene Expression Analysis

We performed quality analysis using fastp (Chen et al., 2018) and FastQC v0.11.7 (Andrews, 2017), and aligned sequences with the reference genome of pepper (Zunla genome) (Qin et al., 2014). Default mapping parameters (10 mismatches/read; nine multi mapping locations/read) were analyzed using HISAT 2.2.1 (Kim D. et al., 2015). We used v1.20.0 of DESeq2, an R-based software package provided by Bioconductor, for differential gene expression analysis (Love et al., 2014).

The abundance of transcripts was measured as the average normalized count of the reads mapped to the transcript, and the difference in their abundance was examined under two conditions to identify transcripts that are differentially expressed under both conditions (Love et al., 2014). The difference in expression was quantified based on the logarithm (change in the logarithmic multiple) of the average normalized count ratio between the two conditions. The differentially expressed transcripts in our experiment were defined as those with adjusted *P*-values < 0.01; cut-off threshold, |log2fold change (FC)| ≥ 2 (negative binomial Wald test, followed by the Benjamini-Hochberg correction). Differentially expressed genes (DEGs) were classified as up-regulated or down-regulated genes based on their significant positive or negative logarithmic changes in value.

Venn diagrams were constructed (Lin et al., 2016). Heatmaps were generated using the seaborn heatmap available in python. Statistical sequencing reads have been included in Supplementary Table 1 and all DEGs genes information in different stages in Supplementary Table 2.

### Co-expression Cluster Recognition Based on the Expression Level of all Genes in Pepper

Co-expression analysis was performed on samples from 12 control tissues and 12 cold stress-treated tissues using the k-means (Gasch and Eisen, 2002) method in python. The normalized expression value of genes was calculated by dividing their expression levels in all samples by their maximum observed transcripts per million (TPM); the cluster information for each expressed gene is displayed in Supplementary Table 3. Principal component analysis (PCA) and hierarchical clustering (HCL) were performed using the Kernel principal component analysis method in python; it was convenient to graphically explain the correlations between all samples using default settings. Transformed normalized gene expression values with *z*-scores were used for PCA and hierarchical clustering.

### Gene Enrichment Analysis

The identified clusters that responded to cold stress were followed up via gene enrichment analysis with GOATOOLS (Klopfenstein et al., 2018), a python package used for gene ontology (GO) enrichment analysis and determination of false discovery rate (FDR) values of statistically significant GO terms (FDR < 0.01). The Kyoto Encyclopedia of Genes and Genomes (KEGG) pathway analysis was performed by KofamKOALA (Aramaki et al., 2020) to protein sequences by homology search, and the enrichment analysis of the KEGG pathway was carried out R package clusterProfiler (Yu et al., 2012; *P* value Cutoff = 0.05).

### Identification of ICE-CBF-COR Signaling Module Genes in Pepper

The *Arabidopsis* genome website contains *Arabidopsis* gene sequence information^1^ and enables us to determine the *Arabidopsis* CBF pathway through a comparison of the protein sequence generated by the *Arabidopsis* gene and the protein sequence generated by the pepper genome gene. Related genes corresponded to orthologous genes in pepper. For important genes in the ICE-CBF-COR-signaling module, the pepper genes with the closest corresponding homology relationship that contain specific structural domains were also identified through homology comparison.

### Expression Analysis of ICE-CBF-COR Signaling Module Genes in Pepper

Fourteen key genes were identified in the ICE-CBF-COR signaling module in pepper, and their expression under control and cold stress-treated conditions was studied. The TPM value of gene expression was normalized using the *z*-score (Supplementary Table 4), and the expression was plotted using a heat map. Then, we used cold tolerance (CT) inbred line A188 and a cold sensitive (CS) inbred line A122 under cold-rewarm treatments and used the RNA-seq data (accession number: PRJNA646356), as described by Miao et al. (2021), to determine the expression levels of the 14 identified genes; then, the *Z*-score was normalized and a heat map was drawn.

### Network Analysis of ICE-CBF-COR Signaling Module Genes in Pepper

We performed Pearson correlation analysis of a total of 29,053 genes that were expressed in at least one sample, obtained the correlation matrix between gene pairs, and extracted 14 CBF pathway-related genes and their correlation information. Python NetworkX enabled us to determine the network relationship diagram between 14 genes.

## Results

### Generation of the Pepper Cold Stress Dataset

**FIGURE 1:**
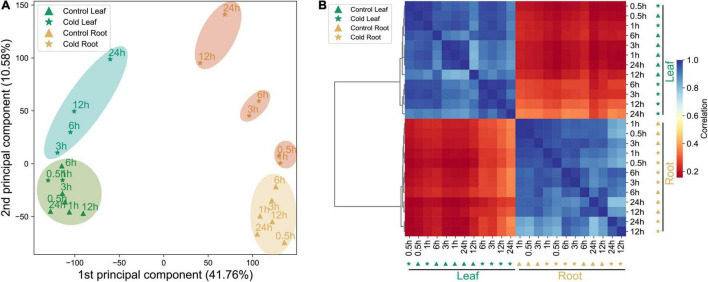
Summary of transcriptome data of the datasets. **(A)** PCA of transcriptome data from the control and cold stress-treated samples (leaf and root). **(B)** The hierarchical clustering analysis of expression profiles of 35,336 genes from 24 samples; the color scale 0–1 represents Pearson’s correlation coefficient.

In order to thoroughly observe the response network of genes in the leaves and roots of pepper plants before and after their exposure to cold stress, we analyzed the complete gene expression in the roots and leaves of control and stress-treated seedlings at six time points, and established a root-based and spatiotemporal dynamic expression data set of whole genes in the leaf tissue. PCA results for the transcriptome showed that the sample exhibited significant tissue specificity (Figure 1A). PCA also showed that different samples can be separated by both PC1 (41.76%) and PC2 (10.58%). In the early stage, when leaves were subjected to cold stress, at 0.5–1 h, the untreated leaves were closer together, and were clustered together at 3–24 h, indicating that the CORs in the leaves of peppers were induced to express proteins 3 h after cold stress treatment. Between 0.5 and 24 h of the root tissue being subjected to cold stress, three main clustering modules could be observed according to the time ranges of 0.5–1, 3–6, and 12–24 h. We circled them with the same colors. The difference in the expression at the root tissues of the three of these and the control showed an increasing trend, and a clear distinction was observed between different treatment times (Figure 1A). It can also be seen that after pepper was subjected to cold stress, the genes in its leaves and roots had strong tissue specificity with regard to the speed and degree of response to cold stress. The Pearson correlation analysis of datasets displayed a gene expression pattern and significant tissue specificity. The same tissues are clustered together (Figure 1B), which is consistent with the phenomenon shown in Figure 1A.

### Pepper Transcriptomes Are Co-regulated in Fifteen Clusters That Correspond to Different Tissues and Treatment Periods

**FIGURE 2:**
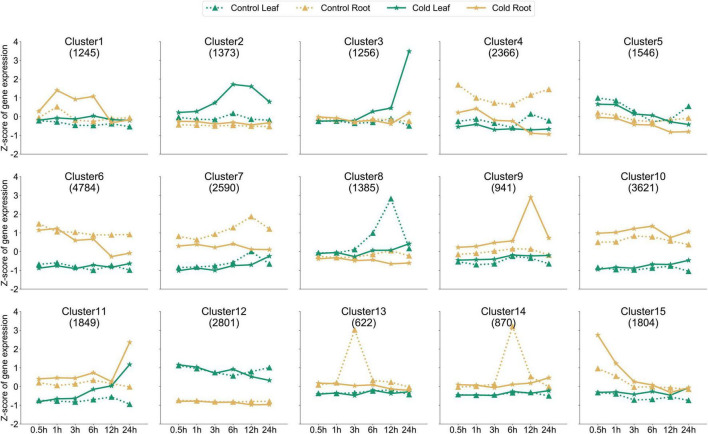
Dynamics of gene expression in all samples. *k*-Means clustering was used to group the expression profiles of the transcriptome into fifteen clusters. The X-axis depicts values at six time points, and the Y-axis depicts the *z*-score standardized per gene. The dotted line represents the roots and leaves of the control, and the solid line represents the roots and leaves of cold stress-treated tissues; different marks represent different organizations. The numbers shown in each box (example: 1,245 genes for cluster 1) indicate the number of genes in that cluster.

In order to further analyze whether the dynamic expression of genes in the roots and leaves of peppers after cold stress treatment exhibits a certain trend of differentiation, we used the k-means clustering algorithm to divide them into at least one sample according to the expression pattern of 29,053 genes. After 15 clusters, the clustering information for each gene is shown in Supplementary Table 3. Upon analyzing these 15 clusters (Figure 2), we found that the expression levels of 1,373 genes in cluster 2 were much higher in the leaf tissues than in the control, 3–24 h after cold stress treatment. The genes in the cluster responded to cold stress. However, the expression levels of genes in cluster 3 were much higher in the leaf tissues under stress at 24 h than those observed in the untreated samples at the same time point when a peak was observed. The expression levels were almost the same at other time points. The 15 clusters formed according to the trend of dynamic expression for the entire gene set had different expression patterns, and gene expression patterns in the same cluster were almost the same. We can classify the COR gene response by time and organization, according to the clustering information. However, we simultaneously found that after cold stress treatment, the expression of some genes in clusters did not change significantly at each sampling time point in the roots and leaves, such as in cluster 5, cluster 10, and cluster 12. Genes may not be sensitive to cold stress, and their expression is always stable.

**FIGURE 3:**
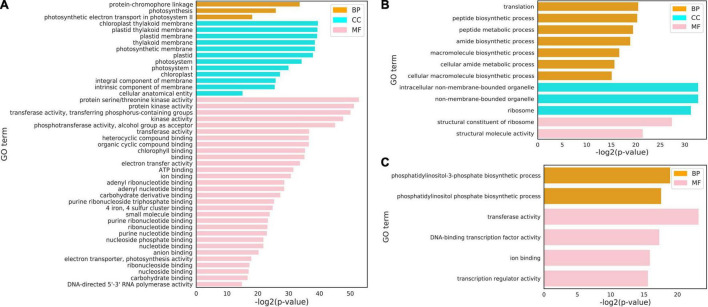
Gene ontology (GO) terms for three clusters under cold stress conditions. **(A–C)** GO terms of cluster 3 (1,256 genes) **(A)**, cluster 9 (941 genes) **(B)** and cluster 15 (1,804 genes) **(C)** all genes at the cellular component (CC), molecular function (MF), and biological process (BP). The complete list of cluster genes and GO terms for enrichment analysis is shown in Supplementary Tables 5–7.

In order to analyze the functional situation of gene clusters with increased expression after cold stress, the three clusters of the cluster 3, cluster 9, and cluster 15 sets were subjected to a GO term enrichment analysis (Figures 3A–C). Those genes in the cluster 3, 9, and 15 were significantly enriched in the “protein-chromophore linkage,” “photosynthesis,” “peptide biosynthetic process,” “amide biosynthetic process,” and “phosphatidylinositol phosphate biosynthetic process” biological processes. The top enriched GO terms in “molecular functions” were related to “protein serine/threonine kinase activity,” “phosphotransferase activity,” “chlorophyll binding,” “structural constituent of ribosome,” “transferase activity,” and “DNA-binding transcription factor activity.” In addition, the top enriched GO terms in “cellular component” were “chloroplast thylakoid membrane,” “plastid thylakoid membrane,” “intracellular non-membrane-bounded organelle,” and “ribosome.”

### Identification of Differentially Expressed Genes Under Cold Stress

**FIGURE 4:**
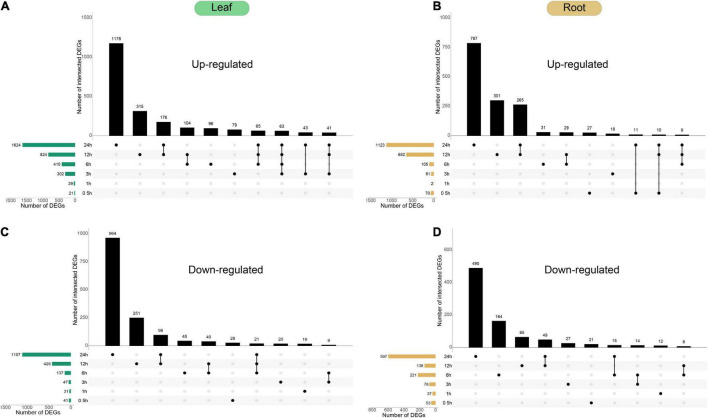
Temporal dynamics of *Capsicum annuum* L. transcriptome during cold treatment. **(A–D)** Forty-day-old *Capsicum annuum* L. plants were subjected to cold stress at 10°C and harvested at the given time points for transcriptome analysis. UpSet plots of the number of up-regulated and down-regulated genes [cut-off threshold, | log2(FC)| ≥ 2; FDR < 0.01] demonstrated different temporal expression patterns (top bar graphs). The total numbers of up-regulated and down-regulated genes at each of the time points are shown on the left.

In order to gain insight into the response mechanism of peppers to cold stress, we conducted a comparative analysis of the transcriptional differences between the leaves and roots treated at 10°C at six time points (Figure 4). DEGs among samples were defined using fold change values, assessed using the expression of assembled transcripts. We have identified 2,306 up-regulated genes and 1,543 down-regulated genes in the leaves of peppers at different processing times. We have also identified 1,551 up-regulated genes and 933 down-regulated genes in the roots; among these, 169 genes exhibited two states of up-regulation and down-regulation in six stress-treated and control leaves. There were 79 genes in the roots in two states, and the remaining DEGs were up-regulated or down-regulated at all periods. Removed genes were identified in both leaves and roots, and the analyzes collectively yielded 4,872 DEGs, constituting ∼16.8% of the expressed genes in the dataset.

In the dynamic situation of gene expression within 0.5–24 h of pepper plants being subjected to cold stress, both tissues had the largest number of DEGs at 24 h. This indicates that a longer duration of exposure to cold stress leads to more profound changes in the pepper transcriptome profile. Currently, there were 1,123 up-regulated genes and 597 down-regulated genes in the roots, and 1,624 up-regulated genes and 1,107 down-regulated genes in the leaves. As shown in Figure 4, most of the differentially expressed genes were unique at 24 h. There were 784 unique up-regulated genes and 490 unique down-regulated genes in the roots, and 1,178 unique up-regulated genes and 964 unique up-regulated genes in the leaves. Among the unique down-regulated genes, the longer the pepper plants were subjected to treatment at 10°C, the greater the change in gene expression at the genome-wide level, and the number of differentially expressed genes in the leaves at 24 h was greater than that in the roots, indicating that pepper leaves have a stronger response to cold stress.

### Functional Enrichment Analysis of Differentially Expressed Genes in the Leaf and Root

**FIGURE 5:**
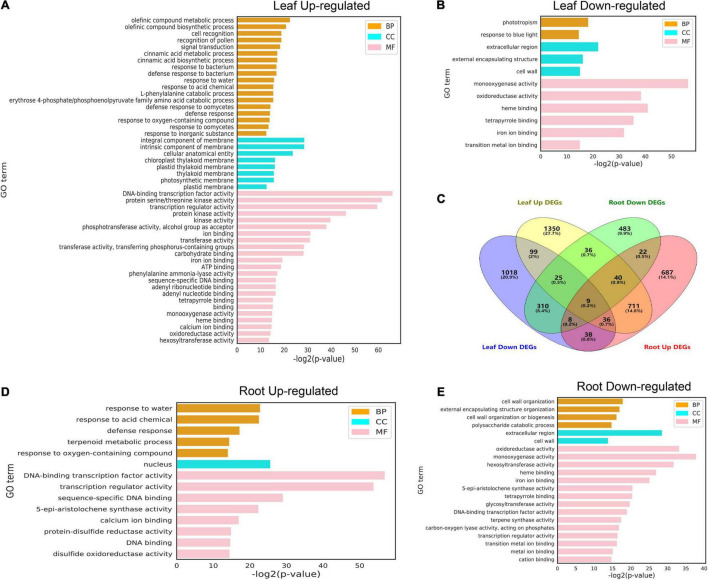
Gene ontology terms for differentially expressed genes (DEGs) under cold stress conditions. **(A,B)** GO terms of upregulated and downregulated DEGs (FDR < 0.01) identified from leaves at different treatment time points. **(C)** Venn diagram of DEGs between the two tissues subjected to cold stress at six different time points. **(D,E)** GO terms of upregulated and downregulated DEGs (FDR < 0.01) identified from roots at different treatment time points. The complete list of DEGs and GO terms for enrichment analysis is shown in Supplementary Tables 8–11.

In order to further analyze the possible functions of the identified cold-responsive genes shown in Figure 4, the up-regulated and down-regulated differentially expressed genes (DEGs) identified in the leaf (Figures 5A,B) and root (Figures 5C,D) were used to perform GO enrichment analysis, and Venn diagrams were used to show the aggregation of up-regulated and down-regulated DEGs in the leaf and root, respectively (Figure 5C). In the leaf and root (Figures 5A,B,D,E), among the “biological process” category, the most significantly enriched terms were “olefinic compound metabolic process,” “phototropism,” “response to water,” and “cell wall organization.” The top enriched GO terms in “molecular functions” were related to “DNA-binding transcription factor activity,” “monooxygenase activity,” “oxidoreductase activity,” and “protein serine/threonine kinase activity.” In addition, the top enriched GO terms in “cellular component” were “integral component of membrane,” “intrinsic component of membrane,” “extracellular region,” and “nucleus.” Low temperatures can slow down the fluidity of cell membranes and stimulate plants to respond to the low temperature.

**FIGURE 6:**
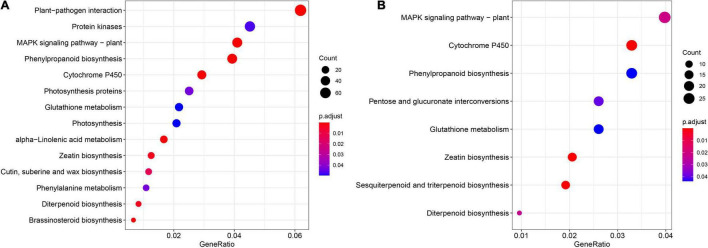
Kyoto encyclopedia of genes and genomes (KEGG) pathway enrichment analysis results for the DEGs in leaf **(A)** and root **(B)** among the six different time points. The complete list of DEGs and KEGG pathways for enrichment analysis is shown in Supplementary Tables 12, 13.

KEGG pathway enrichment was carried out in order to better understand the biological functions of cold stress DEGs in leaf and root. The enrichment analysis indicated that “MAPK signaling pathway,” “Phenylpropanoid biosynthesis,” “Cytochrome P450,” “Glutathione metabolism,” “Zeatin biosynthesis,” and “Diterpenoid biosynthesis” were common in leaf and root (Figure 6). However, “Plant-pathogen interaction”, “Protein kinases,” “Photosynthesis proteins,” “alpha-Linolenic acid metabolism,” “Cutin, suberine and wax biosynthesis” and “Brassinosteroid biosynthesis” were unique in leaf (Figure 6A) and “Pentose and glucuronate interconversions” and “Sesquiterpenoid and triterpenoid biosynthesis” were unique in root (Figure 6B).

### Identification and Functional Analysis of Pepper ICE-CBF-COR Signaling Module Genes

**FIGURE 7:**
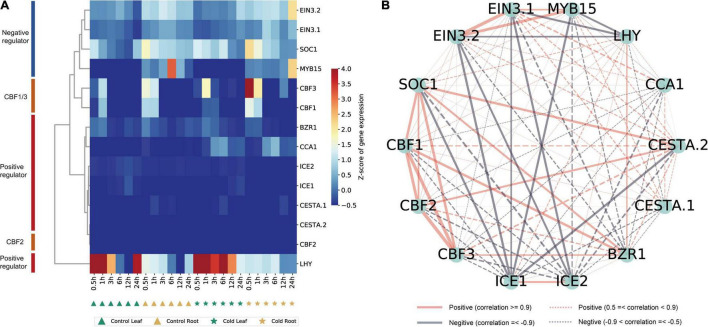
Identification of ICE-CBF-COR genes in pepper and visualization of their co-expression network. **(A)** Hierarchical cluster analysis of 14 ICE-CBF-COR genes. The values in the heatmap represent the *z*-scores of transcripts per million (transcription level) in different samples. The red and blue colors indicate a high and low expression level, respectively. **(B)** Visualization of the 14 ICE-CBF-COR gene co-expression networks using the Python NetworkX package.

Several studies have focused on ICE-CBF-COR in *Arabidopsis* and some model plants (Thomashow, 1999; Chinnusamy et al., 2003; Kim Y. S. et al., 2015; Barrero-Gil and Salinas, 2017). The ICE-CBF-COR-related genes in peppers have not been systematically identified and functionally analyzed. The expression of ICE-CBF-COR-related genes in peppers under low-temperature stress and their regulatory relationships needed to be examined. Therefore, based on the rich research background associated with ICE-CBF-COR in *Arabidopsis*, and based on the conservation of ICE-CBF-COR in the plant and protein sequence alignment, we identified that pepper contains 14 *CBF1/2/3*-related genes, including *ICE1/2* and *ICE1/2*, and *MYB15*, which have been thoroughly studied in *Arabidopsis* (Agarwal et al., 2006), *SOC1*, positive regulators of *EIN3*, *Brassinazole-resistant1* (*BZR1*) (Li et al., 2017b), *CCA1*, *CESTA* (*CES*) (Eremina et al., 2017), and positive regulators of *LHY* (Seo et al., 2009; Dong et al., 2011; Lee and Thomashow, 2012; Eremina et al., 2016). Notably, the normalized data for the control tissues obtained from the leaves and roots of pepper included that for the 14 orthologous genes in pepper and six time points at which transcript levels observed after cold stress enabled us to distinguish between negative regulators and positive regulators (Figure 7A). In *Arabidopsis*, the expression of *CBF1* and *CBF3* in response to a low temperature precedes that of *CBF2*, and *CBF2* negatively regulates *CBF1* and *CBF3* expression (Novillo et al., 2004). In our pepper dataset, we can also see that *CBF1* and *CBF3* were clustered together, but the distance from *CBF2* was high, indicating that the gene functions of *CBF1/2/3* were conserved in pepper, implying that there is a certain degree of conservation in *A. thaliana*. Genes that are negatively or positively regulated by *CBF* also have similar effects in pepper, which is of great value for the study of the ICE-CBF-COR signaling module in pepper.

**FIGURE 8:**
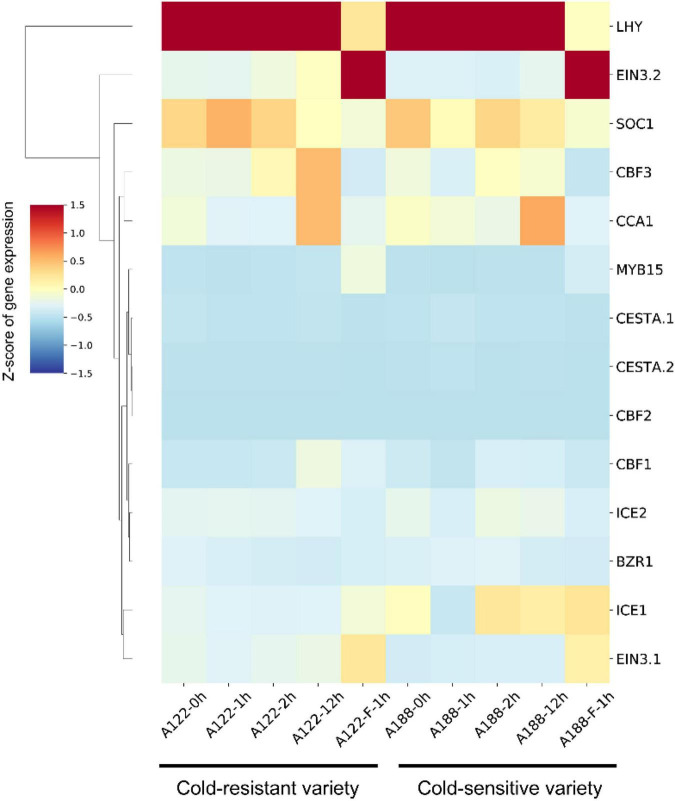
Hierarchical cluster analysis of 14 ICE-CBF-COR genes in A122 and A188. The values in the heatmap represent the *z*-scores of fragments per kilobase million (transcription level) in different samples. The red and blue colors indicate high and low expression levels, respectively.

We used this data set to establish a co-expression regulatory network of these 14 genes in pepper and visualized the mutual regulatory relationship between these 14 genes. The gray lines represent negative regulatory relationships between genes, and the red lines represent the positive regulatory relationship between genes. Positive regulatory relationships have also been shown. The increased thickness of the line represents a stronger predicted relationship, and the dotted line represents the Pearson coefficient absolute value being less than 0.5. We found that the relationship predicted in pepper is the same as that observed in *A. thaliana* (Figure 7B). For example, *MYB15*, *SOC1*, and *EIN3* negatively regulate *ICE1* expression in *Arabidopsis* (Agarwal et al., 2006). The same result is also shown in our network diagram. The co-expression relationship between these 14 genes can provide certain insights into the correlation between related genes in pepper and the ICE-CBF-COR signaling module at low temperatures. Furthermore, we used predecessors in the cold-resistant (A122) and cold-sensitive (A188) varieties of pepper under cold stress (4°C for 0, 1, 2, and 24 h) conditions and after rewarming (28°C for 1 h), and obtained the RNA-seq data (Miao et al., 2021). We analyzed the expression of the 14 genes identified in the dataset and found that after the rewarming process, the expression of *EIN3.1* and *EIN3.2* was significantly up-regulated, and the expression of *ICE1/2* in A188 was significantly higher than that in A122 (Figure 8).

## Discussion

Owing to the completion of the assembly of the whole genome of pepper (Kim et al., 2014; Qin et al., 2014). The whole-genome sequencing of pepper has made it feasible to identify stress resistance genes in the pepper plant, at the genome level. Cold weather often leads to a severe decline in crop yields (Zhang J. et al., 2019). Although the molecular mechanism of cold-induced reprogramming of gene expression has been studied extensively in model plants, only a few of these reports are related to peppers (Liu et al., 2021; Miao et al., 2021). In this study, we tried to determine the regulatory mechanism of the response to cold stress in pepper by subjecting leaves and roots to cold stress at six time points and performing transcriptome analysis of the stress-treated and control tissues at each time point. Cold acclimation involves a cascade of transcriptional events. We divided all samples into 15 clusters based on the expression pattern of 29,053 genes in at least one sample, and found that the expression levels of genes in cluster 2 and cluster 3 in the leaves subjected to cold stress were much higher at different time points than those of the control. For example, the expression of 1,373 genes in cluster 2 was higher at 0.5 h, and the peak difference was observed at 6 h, while the expression of 1,256 genes in cluster 3 increased sharply at 24 h. Further, the cluster 3, cluster 9, and cluster 15 were enriched in different GO terms (Figures 3A–C). In this study, many tissue-specific DEGs were identified in leaf and root six time points, which may be due to the different effects of cold stress on the leaf and root of pepper seedlings (Figures 5A–D). However, these tissue-specific DEGs are also enriched in pathways, such as the “MAPK signaling pathway”, suggesting that it plays a key role in the transduction of environmental and developmental signals (Figures 6A,B; Jagodzik et al., 2018) and previous studies have reported that *A. thaliana* and rice (*Oryza sativa*) participate in the response to freezing stress through the MAPK cascade pathway (Li et al., 2017a; Zhang et al., 2017; Zhao et al., 2017).

Moreover, we identified 14 genes in the pepper ICE-CBF-COR signaling module, and analyzed the expression and clustering of these genes in different tissues and time points under cold stress (Figure 7A). We found that positively and negative regulatory factors in pepper are clustered together, which is consistent with the results of the study of *A. thaliana*, which proves that ICE-CBF-COR is conserved in plants (Kim Y. S. et al., 2015).

We combined the RNA-seq data for the cold-resistant (A122) and cold-sensitive (A188) varieties identified previously after low-temperature treatment and rewarming, to verify the results of our analysis (Miao et al., 2021). We found that the expression level of positive regulators, such as *ICE1/2*, *CBF1*, and *CCA1* was higher in A188 than in A122. After being subjected to cold stress, the stress response in A122 material was stronger, which may be attributable to its significantly lower tolerance to cold stress than A188, as a higher level of expression of these positive regulators was needed to activate the ICE-CBF-COR-signaling module, and activate low-temperature tolerance in the plant more effectively. In addition, we found that the expression levels of *EIN3.1/2*, *MYB15*, and *ICE1* increased in A122 and A188 after rewarming. In *Arabidopsis*, cold stress up-regulates the expression of *MYB15*, and the *MYB15* protein interacts with *ICE1* and binds to the MYB recognition sequence in the promoter sequence of CBFs (Agarwal et al., 2006). The performance of *MYB15* in pepper is different. In the normal pepper variety 6421, the expression of *MYB15* in the root increases under cold stress at 24 h, but there is no significant response in the leaves (Figure 7A). The role of *MYB15* in *A. thaliana* is supported by data showing that *MYB15* overexpression and low expression alter the expression of CBF genes and affect freezing tolerance (Agarwal et al., 2006). The response of *MYB15* in pepper A122 was stronger than that in A188, which also confirmed the situation in *Arabidopsis*.

In this study, we analyzed the transcriptome data for cold stress at six time points in the leaves and roots of the pepper plant, determined the co-expression-based relationship between 14 genes in the pepper ICE-CBF-COR-signaling module, and correlated it with that in A122, which had been identified by previous researchers (Miao et al., 2021). The results of transcriptome analysis of extreme low temperature-tolerant tissues of A188 showed that the expression of these genes occurred in cold-resistant and cold-sensitive varieties after they were subjected to low-temperature stress and rewarming (Figure 8). These results provide another perspective regarding the low-temperature reaction mechanism in pepper and *Solanaceae* crops. In this study, we not only identified the key genes in the ICE-CBF-COR-signaling module in pepper, but also constructed a mutual co-expression network of these genes based on the low-temperature stress data set for the leaf and root at six time points. This network needs to be verified via experiments involving genetic transformation in the future, to verify the response mechanism of pepper to low temperatures. In this study, we have used bioinformatics tools to enable follow-up researchers to perform verification in a more effective manner.

## Conclusion

In this study, we performed a global analysis of 24 samples of leaves and roots of peppers at six time points after subjecting them to low-temperature stress. Control treatments were also performed to assess the dynamic expression of different tissues in peppers at the whole gene level subjected to low temperature stress for different durations. We identified 3,680 and 2,405 DEGs in the leaves and roots, respectively. Fourteen low-temperature-responsive ICE-CBF-COR modular genes were identified in peppers, and a pairwise co-expression network model was constructed between the 14 genes. The expression patterns in different pepper varieties showed that they were resistant to cold stress. The process of collection of data in this study under low-temperature stress would act as a reference for the exploration of a plant ICE-CBF-COR signal model, and contribute to the elucidation of the response mechanism to low temperatures and adaptability to other stress conditions.

## Data Availability Statement

The datasets presented in this study can be found in online repositories. The name of the repository and accession number can be found below: National Genomics Data Center (NGDC), China National Center for Bioinformation (CNCB)/Beijing Institute of Genomics (BIG), Chinese Academy of Sciences (CAS), Genome Sequence Archive (GSA), https://ngdc.cncb.ac.cn/gsa/, PRJCA007952.

## Author Contributions

LX and XL: conceptualization. BT: data curation, visualization, and writing – original draft. FL, XD, and XZ: funding acquisition. HY and YC: investigation. BT and FL: writing – review and editing. All authors contributed to the article and approved the submitted version.

## Conflict of Interest

The authors declare that the research was conducted in the absence of any commercial or financial relationships that could be construed as a potential conflict of interest.

## Publisher’s Note

All claims expressed in this article are solely those of the authors and do not necessarily represent those of their affiliated organizations, or those of the publisher, the editors and the reviewers. Any product that may be evaluated in this article, or claim that may be made by its manufacturer, is not guaranteed or endorsed by the publisher.

## Footnotes

1
https://www.arabidopsis.org/

## Abbreviations

DEGs: differentially expressed genes
CORs: cold-responsive genes
RNA-seq: RNA sequence
HPT: hours post-treatment
TPM: transcripts per million
FPKM: fragments per kilobase million
HCL: hierarchical clustering
PCA: principal component analysis
FDR: false discovery rate
FC: fold change
GO: gene ontology
BP: biological process
MF: molecular function
CC: cellular component
KEGG: kyoto encyclopedia of genes and genomes
CT: cold tolerance
CS: cold sensitive.
